# Neurobiological links between stress and anxiety

**DOI:** 10.1016/j.ynstr.2019.100191

**Published:** 2019-08-13

**Authors:** Nuria Daviu, Michael R. Bruchas, Bita Moghaddam, Carmen Sandi, Anna Beyeler

**Affiliations:** a Hotchkiss Brain Institute. Department of Physiology & Pharmacology, Cumming School of Medicine, University of Calgary, 3330 Hospital Drive NW, Calgary, AB, T2N 4N1, Canada; b Department of Anesthesiology and Pain Medicine. Center for Neurobiology of Addiction, Pain, and Emotion. University of Washington. 1959 NE Pacific Street, J-wing Health Sciences. Seattle, WA 98195, USA; c Department of Behavioral Neuroscience, Oregon Health and Science University, Portland, OR, 97239, USA; d Laboratory of Behavioral Genetics, Brain Mind Institute, École Polytechnique Fédérale de Lausanne (EPFL), Station 19, CH, 1015, Lausanne, Switzerland; e Neurocentre Magendie, INSERM 1215, Université de Bordeaux, 146 Rue Léo Saignat, 33000 Bordeaux, France

**Keywords:** Neural circuits, Optogenetics, Mitochondria, Corticotrophin releasing hormone, Emotional valence

## Abstract

Stress and anxiety have intertwined behavioral and neural underpinnings. These commonalities are critical for understanding each state, as well as their mutual interactions. Grasping the mechanisms underlying this bidirectional relationship will have major clinical implications for managing a wide range of psychopathologies. After briefly defining key concepts for the study of stress and anxiety in pre-clinical models, we present circuit, as well as cellular and molecular mechanisms involved in either or both stress and anxiety. First, we review studies on divergent circuits of the basolateral amygdala (BLA) underlying emotional valence processing and anxiety-like behaviors, and how norepinephrine inputs from the locus coeruleus (LC) to the BLA are responsible for acute-stress induced anxiety. We then describe recent studies revealing a new role for mitochondrial function within the nucleus accumbens (NAc), defining individual trait anxiety in rodents, and participating in the link between stress and anxiety. Next, we report findings on the impact of anxiety on reward encoding through alteration of circuit dynamic synchronicity. Finally, we present work unravelling a new role for hypothalamic corticotropin-releasing hormone (CRH) neurons in controlling anxiety-like and stress-induce behaviors. Altogether, the research reviewed here reveals circuits sharing subcortical nodes and underlying the processing of both stress and anxiety. Understanding the neural overlap between these two psychobiological states, might provide alternative strategies to manage disorders such as post-traumatic stress disorder (PTSD).

## 1 Introduction

Although the relationship between psychological stress and anxiety seems intuitive, the biological nuances that distinguish the two states are extremely complex. Indeed, after decades of research in psychology, ethology and neurophysiology, overlapping neural substrates of these two psychobiological states have been identified. However, the boundaries between stress and anxiety remain an open discussion.

A stress response, created by a real or perceived threat (stressor), can be defined as an emergency state of an organism in response to a challenge to its homeostasis (Chrousos, 2009; Selye, 1936). During this emergency state, the organism initiates an integrated reaction including physiological and behavioral responses. Internal threats, or so-called systemic stressors, include physical changes in the body, such as hypoglycemia or hypovolemia (decreased blood volume), happening, for example, after a severe car accident. On the other hand, perceived threats, or so-called psychological stressors, include situations that can potentially lead to a danger and induce a homeostatic challenge, introducing the critical factor of *anticipation* (de Kloet et al., 2005; Koolhaas, 2011). The concept of anticipation in the stress response is critical in understanding the relationship between stress and anxiety. In that regard, stress as a physiological reaction to a stimulus is accompanied by a concomitant emotional response. That emotional response is determined in part by the perception of the threat imminence (Anderson and Adolphs, 2014; Davis et al., 2010). According to the definition of *The Diagnostic and Statistical Manual of Mental Disorders, Fifth Edition* (DSM-5) (American Psychiatric Association, 2013) “*Fear* is the emotional response to a real or perceived imminent threat, whereas *anxiety* is the anticipation of a future threat”. Thus, the emotional state that our body experiences differs between fear when we encounter an aggressive dog, and anxiety when we know we will visit a friend which has an aggressive dog.

Anxiety is defined as a temporally diffused emotional state caused by a potentially harmful situation, with the probability or occurrence of harm being low or uncertain (Goes et al., 2018; Spielberger et al., 1983; Takagi et al., 2018). Historically, psychologists and psychiatrists have differentiated state and trait anxiety (Belzung and Griebel, 2001; Goes et al., 2018; Spielberger et al., 1983; Takagi et al., 2018). The diverging element of these two types of anxiety is their duration: state anxiety is an acute response to a potential threat, while trait anxiety is chronic, as it is expressed constantly during the life of the individual, and is therefore considered as a trait of an individual's personality (Endler and Kocovski, 2001; Spielberger et al., 1983). State anxiety can be defined as hypervigilance in anticipation of a threat that can be triggered by acute stress, and has the primary function of avoiding dangerous situations and also to facilitate memory consolidation (Roozendaal et al., 2008). On the other hand, trait anxiety is a predisposition of an individual to express constant anxiety, and increases the probability of state anxiety in potentially dangerous situations (Endler and Kocovski, 2001; Spielberger et al., 1983). State and trait anxiety are not mutually exclusive, and state anxiety triggered by an event can be superimposed on trait anxiety. Importantly, both state and trait anxiety responses represent an evolutionary advantage to anticipate and avoid danger (Goes et al., 2018; Spielberger et al., 1983; Takagi et al., 2018). Therefore, anxiety *per se* is not a pathological state, as it can prevent exposure to dangerous situations. However, when anxiety is sustained and/or elicited by non-threating stimuli, it becomes maladaptive (Belzung and Griebel, 2001; Sylvers et al., 2011). While state and trait anxiety are essential psychological metrics to evaluate normal and pathological levels of anxiety, these metrics do not consider the neural substrate of anxiety, which partly explains the lack of new and effective therapies for anxiety disorders.

Over the past twenty years, human functional imaging has identified multiple brain areas including the hypothalamus, amygdala, prefrontal cortex and nuclei of the brainstem which are active during both stress and anxiety responses in healthy individuals (Mobbs et al., 2007; Takagi et al., 2018). Interestingly, a subset of brain regions including the basolateral amygdala (BLA), medial prefrontal cortex (mPFC), locus coeruleus (LC), as well as reward processing areas such as the nucleus accumbens (NAc), appear to be affected in animal models of both stress disorders and anxiety disorders (Calhoon and Tye, 2015; Etkin and Wager, 2007; Sailer et al., 2008; Shin and Liberzon, 2010). The intermingled neural circuits controlling both stress and anxiety suggests a strong bidirectional relationship between stress experiences and anxiety in both healthy and pathological conditions. Therefore, alterations of the connectivity between the brain regions influencing both stress and anxiety behaviors might contribute to the etiology of psychopathologies such as generalized anxiety disorder (GAD), social anxiety disorders or post-traumatic stress disorder (PTSD).

Herein, we summarize the views of the panel on *Stress, Anxiety and Corticolimbic Pathways*, presented at the 2018 Stress Neurobiology meeting, held in Banff, Canada. This perspective reflects the diverse and shared structures involved in both stress and anxiety responses. We aim to reveal common subcortical processes that could support the interplay between stress and anxiety-related behaviors.

## 2 Coding of emotional valence in the basolateral amygdala (BLA)

The attribution of emotional valence to sensory information is a key process that allows individuals to navigate the world, and has been shown to be altered in both anxiety and stress disorders (Etkin and Wager, 2007; Sailer et al., 2008). Valence is the subjective value assigned to sensory stimuli, which determines subsequent behavior. Positive valence leads to approach and consummatory behaviors while negative valence leads to defensive and avoidance behaviors (Pignatelli and Beyeler, 2019; Russell, 1980). Attentional bias for stimuli of negative valence have been extensively demonstrated in patients with anxiety disorders (MacLeod et al., 2019). For example, anxiety increases negative interpretations of ambiguous sentences (Richards and French, 1992) and scenarios (Hirsch and Mathews, 1997) suggesting an anxiety-induced valence bias. Recent publications showed that high trait anxiety individuals exhibit a bias towards negative interpretations of surprised faces (Park et al., 2016a). The existence of a correlation between negative valence bias and the level of anxiety in health and disease observed in human studies supports the hypothesis that the circuits encoding emotional valence could be dysfunctional in anxiety disorders (Etkin and Wager, 2007; Lammel et al., 2014; Mervaala et al., 2000; Pignatelli and Beyeler, 2019). A key structure encoding emotional valence and therefore guiding animal behavior is the basolateral nucleus of the amygdala (BLA). This region receives sensory inputs of multiple modalities, and projects to output structures controlling behavioral responses (Janak and Tye, 2015; McDonald, 1998). This central connectivity has made the BLA a focus for identifying the neural substrate of valence processing. Moreover, a vast body of literature has shown that BLA integrity is critical for processing positive (Bucy and Klüver, 1955; Tye et al., 2008; Weiskrantz, 1956) and negative valence (Bucy and Klüver, 1955; LeDoux et al., 1990; McDonald, 1998; Tye et al., 2008; Weiskrantz, 1956).

**Fig. 1:**
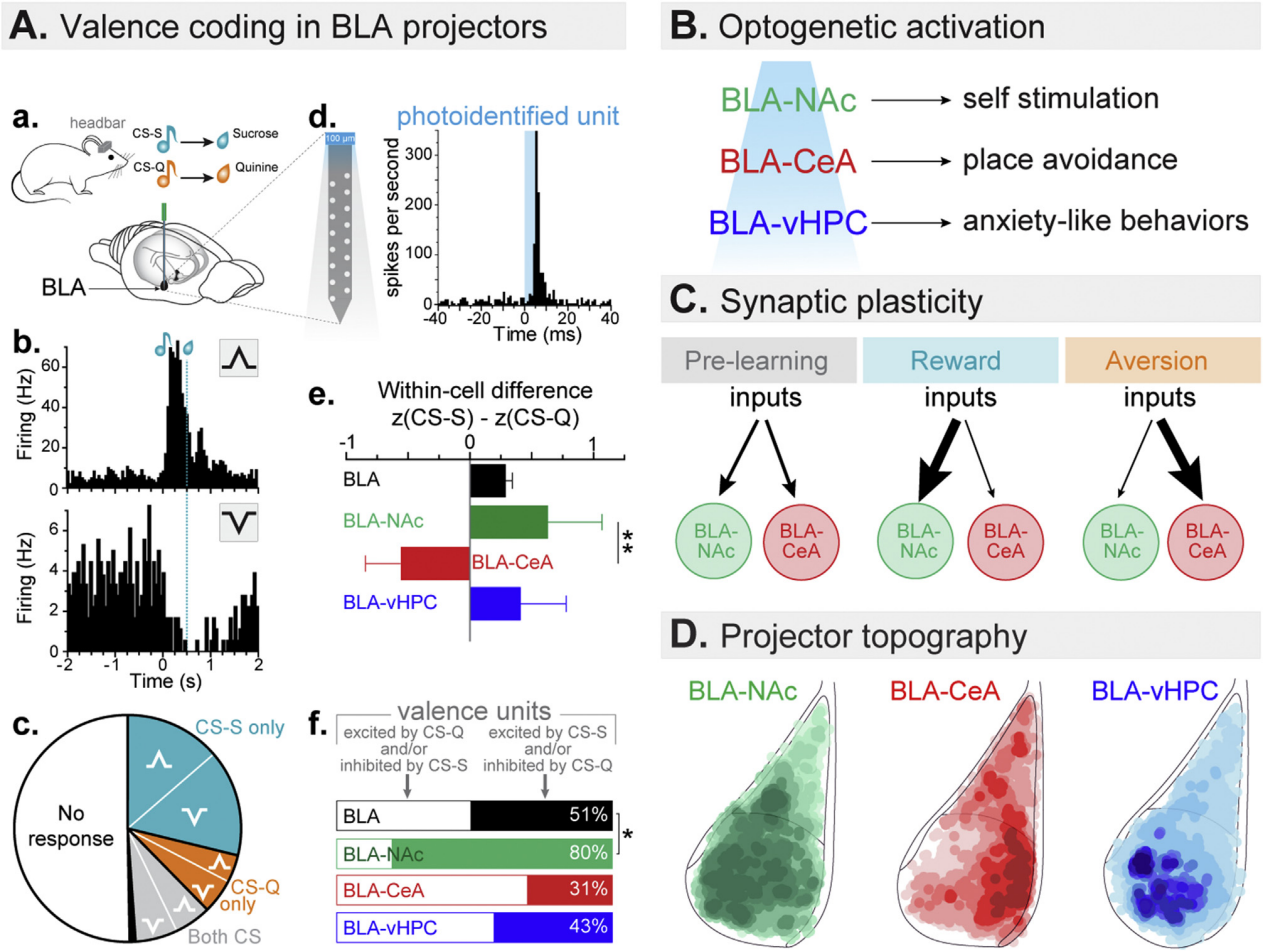
**Valence coding in the basolateral amygdala (BLA) projector populations. A.** Projector valence coding (adapted from Beyeler et al. 2016). **a.** Schematic of Pavlovian conditioning paradigm. Head-fixed mice were trained to discriminate between one cue paired with sucrose (CS–S) and a different cue paired with quinine (CS-Q). **b.** Peri-stimulus time histogram (PSTH) of the firing rates of representative units excited (top) or inhibited (bottom) during a CS-S presentation followed by a sucrose delivery. **c.** Fraction of BLA neurons excited or inhibited by CS-S, CS-Q or both. **d.** PSTH of action potentials of a BLA single-unit photoidentified as a BLA-NAc projector. **e.** Within-cell difference of response to CS-S and CS-Q depending on the neurons projection targets. **f.** Percentage of positive and negative valence units in the BLA. **B.** Behavioral impact of optogenetic activation of different BLA pathways (BLA-NAc and BLA-CeA projectors and BLA-vHPC terminals). **C.** Synaptic plasticity mechanism observed in BLA-NAc and BLA-CeA projection neurons after learning of valence associations. **D.** Topographic maps of three projectors populations in the BLA. CS: conditioned stimuli, S: sucorse, Q: quinine, NAc: nucleus accumbens, CeA: central amygdala, vHPC: ventral hippocampus.

Interestingly, studies have shown that when defined by their projection target, neurons of the BLA differentially control and encode emotional valence (Fig. 1A). A set of studies revealed that photostimulation of BLA neurons synapsing in the medial core/shell section of the NAc (BLA-NAc) support reward seeking (Namburi et al., 2015). Meanwhile, BLA neurons projecting to the medial section of the central amygdala (BLA-CeA) mediate place avoidance (Namburi et al., 2015). Importantly, these two different BLA populations have divergent responses to valence predicting cues. Indeed, single-unit *in vivo* recordings combined with optogenetic photoidentification indicated that a higher proportion of BLA-NAc neurons were excited by a cue predicting a reward, while BLA-CeA neurons showed a higher proportion of neurons excited by a stimulus predicting an aversive outcome (Beyeler et al., 2016).

Furthermore, the authors identified a synaptic mechanism for learning of valence associations. Specifically, synaptic inputs onto BLA-NAc neurons and BLA-CeA neurons undergo opposing synaptic changes following reward or fear conditioning (Fig. 1C, Namburi et al., 2015). Notably, BLA neurons projecting to those distinct areas are intermingled within the BLA, but are distributed following topographical gradients (Fig. 1D, Beyeler et al., 2018), which are correlated with a dorso-ventral bias of negative to positive valence coding (Beyeler et al., 2018).

Another set of optogenetic experiments have shown that activation of the BLA projection to the ventral hippocampus (vHPC) is sufficient to induce real time anxiogenic effects and conversely, inhibition of those projections causes an anxiolytic effect (Fig. 1B, Felix-Ortiz et al., 2013). Single-unit recordings combined with optogenetic photoidentification have shown that BLA-vHPC neurons have no coding bias for learned stimuli predicting outcomes of positive or negative valence compared to the entire BLA (Beyeler et al., 2016). This observation supports the idea that BLA-vHPC neurons may mediate anxiety-related behaviors which can be defined as an innate state of negative valence, rather than learned valence.

Altogether, the BLA is a single structure which includes neural populations that underlie processing of learned emotional valence (BLA-NAc and BLA-CeA) and a population that generates innate emotional states (BLA-vHPC). This finding suggests that BLA is a key structure to study how emotional states such as anxiety interfere with emotional valence processing.

## 3 Locus coeruleus noradrenergic (LC-NE) projections to BLA: acute stress-induced anxiety

Stressful experiences engage multiple structures to generate a coordinated physical and psychological response to a challenge. The locus coeruleus noradrenergic system (LC-NE) presents a brain-wide projection pattern (Schwarz et al., 2015) and is linked to both physical and emotional responses to stress (Berridge and Waterhouse, 2003; Valentino and Van Bockstaele, 2008), as well as aversive memory consolidation (Roozendaal et al., 2008). Noradrenaline release during an acute stress induces a state anxiety response that allow the organism to maintain high attention, facilitate sensory processing and enhance executive functions in order to increase memory consolidation during stressful experiences (Berridge and Waterhouse, 2003; Sara and Bouret, 2012).

Electrophysiological and optogenetic studies indicate that LC-NE neurons normally display three activation profiles: low tonic (1–2 Hz), high tonic (3–8 Hz) and phasic activity (Carter et al., 2010). Acute stress causes a robust increase in tonic firing rate in LC-NE (Valentino and Van Bockstaele, 2008) and this stress-induced tonic firing is associated with an increase in anxiety-like behavior. The role of tonic activation of LC-NE neurons is further supported by the observations that optogenetic stimulation of NE cell bodies in the LC, in the absence of a stressor, mimics LC-NE tonic activity as well as acute-stress induced anxiety (McCall et al., 2015). Furthermore, they suggest that this increase of activity in LC-NE neurons is caused by synaptic inputs into the LC containing corticotropin releasing hormone (CRH^+^). Specifically, stimulation of CRH^+^ CeA-LC terminals increases activity in LC and drives anxiety-like behaviors through type 1 CRH receptor (CRH1R) activation (McCall et al., 2015).

Acute stress also promotes anxiety and other stress related behaviors through BLA adrenergic receptor activation (Chang and Grace, 2013). Even though the anatomical projections from LC and the role of NE in stress and anxiety have been studied extensively, the mechanism by which LC-NE influences BLA function to promote negative emotional states has only recently been unravelled. McCall and collaborators (2017) demonstrated that optogenetic activation of LC-NE fibers in the BLA in acute brain slices causes norepinephrine release into the BLA. *In vivo* photostimulation of these terminals modulates BLA activity, and LC-BLA stimulation is sufficient to cause conditioned place aversion as well as anxiety-like behaviors. These stimulation-induced behavioral changes require β-adrenergic receptor activity in the BLA, providing *in vivo* evidence that endogenous NE release from LC terminals alters BLA function and, as a consequence, modifies behavior. Additional support for β-adrenergic signaling promoting anxiety-like behaviors is provided in Siuda et al. (2015), whereby selective optical activation of β-adrenergic signaling in CaMKII(+) neurons of the BLA produces robust anxiety-like phenotypes.

**Fig. 2:**
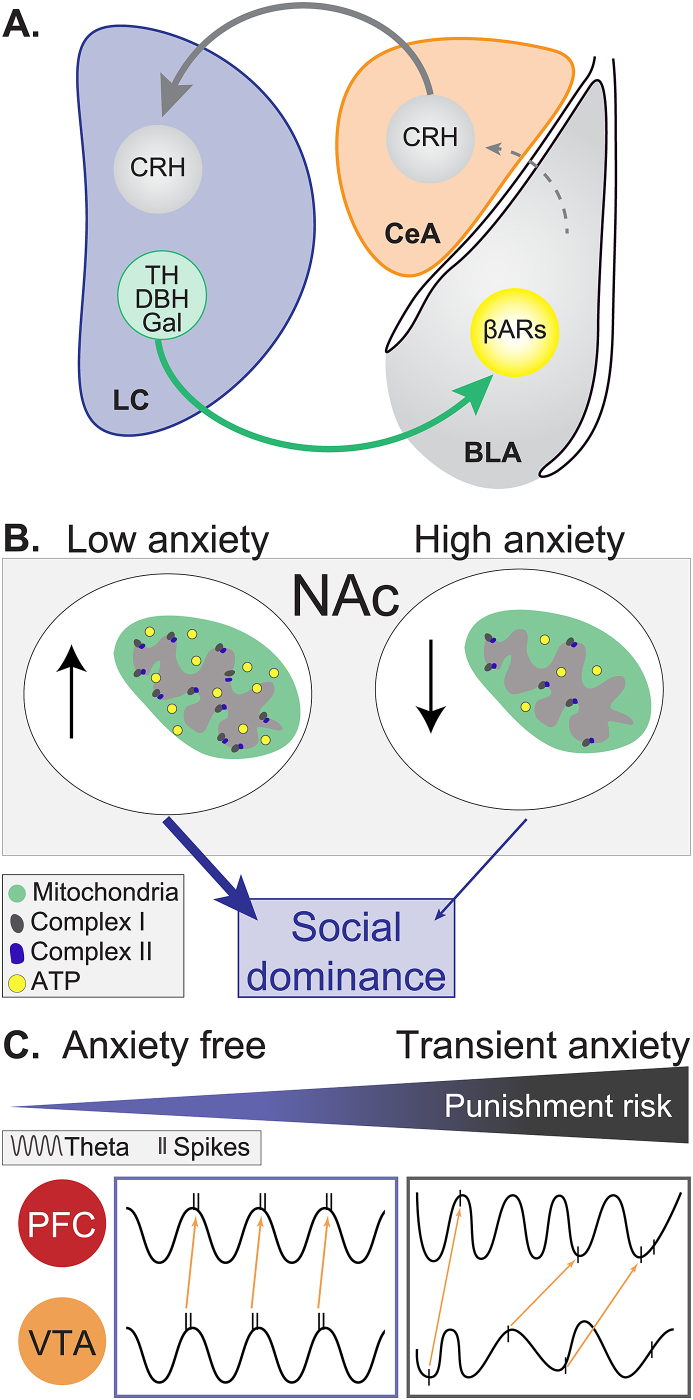
**Circuit and molecular mechanisms of stress and anxiety. A.**LC-NE projections to BLA increases anxiety-like behaviors acting on β-adrenergic receptors (βARs) and through projections to downstream structures such as the CeA. **B.**NAc mitochondrial function and anxiety, and its influence on social dominance. **C.**Circuit synchronicity between the PFC and VTA under different punishment probabilities. LC: locus coeruleus, BLA: basolateral amygdala, CeA: central amygdala, NAc: nucleus accumbens, PFC: prefrontal cortex, VTA: ventral tegmental area, NE: norepinephrine; ATP: adenosine triphosphate CRH: corticotropin-releasing hormone TH: tyrosine hydroxylase DBH: dopamine beta-hydroxylase Gal: galanin.

LC-NE neurons preferentially target neurons in the BLA that project to the ventral hippocampus (BLA-vHPC) and CeA (BLA-CeA), both downstream structures involved in negative valence and anxiety-related behaviors (Beyeler et al., 2018; Felix-Ortiz et al., 2013; Namburi et al., 2015). This suggests that LC-NE projections to BLA increase anxiety-like behaviors following stress exposure, through projections to downstream structures such as CeA or vHPC (Fig. 2A). This recent work reveals that part of the circuit underlying acute-stress, induces state anxiety.

### 3.1 A new mitochondrial function linking stress and emotional traits

Even in our modern society, the necessity of positioning ourselves in a social group through social competition has an enormous impact on our daily lives. In spite of the importance that social competition has in organizing and structuring our society, the psychological characteristics that affect social competitiveness of an individual have been largely overlooked. Several brain regions such as the amygdala and the NAc have been implicated in social status and competition in both humans (Zink et al., 2008), and rodents (Goette et al., 2015). Recent studies have specifically revealed the critical role of the NAc in social competition and the establishment of social status (Hollis et al., 2015; Larrieu et al., 2017; Van der Kooij et al., 2018). During social competition, D1-containing medium spiny neurons (MSNs) in the NAc are activated and show a positive correlation with the level of offensive behavior observed in a social hierarchy test. In addition, when the NAc, but not the BLA, is inactivated with a GABA_A_ receptor agonist during a social competition test, rats showed reduced social dominance (Hollis et al., 2015).

The NAc is involved in motivation and has been implicated in the regulation of anxiety and depressive-like symptoms (Lüthi and Lüscher, 2014). The neural mechanism through which anxiety might affect social hierarchy has been poorly investigated. In humans, high-anxiety individuals tend to display subordinate roles and to be less competitive in social environments (Gilbert et al., 2009). Likewise, high-anxiety rats show less social dominance after a social competition for a territory (Hollis et al., 2015). These results are consistent with data obtained in humans where high anxiety traits predispose subjects for social submission (Goette et al., 2015). These behavioral results, together with the new data revealing a unique role of the NAc in the establishment of social hierarchy open a new path to investigating how NAc function can bridge anxiety and social competition. In search of potential mechanisms within the NAc, which could differentiate low and high anxiety rats, Hollis et al. (2015) showed that high anxiety rats had lower mitochondrial activity in NAc, compared to low anxiety rats. Specifically, with similar mitochondrial numbers and density, highly anxious rats have lower levels of respiratory complexes I and II of the electron transport chain, resulting in a reduced mitochondrial function (Fig. 2B). Furthermore, social status also predicts behavioral stress susceptibility and metabolic profile in the NAc after chronic social defeat (Larrieu et al., 2017). These studies establish a key role of mitochondrial function in individual differences that impact social dominance in a non-pathological condition. Altogether, the newly discovered role of mitochondrial energy metabolism in the NAc in anxiety-induced social deficits opens a new path for therapeutic treatment that targets cell metabolism.

In regards to the relationship between stress and anxiety, social competition itself induces an endocrine stress response (Turan et al., 2015). In humans, stress exposure differentially affects low and high anxiety subjects in a competitive task. Under stressful conditions, low-anxiety individuals become overconfident, while high-anxiety individuals show less social confidence (Goette et al., 2015). Moreover, several studies have reported increased risk of adult psychopathologies after early life adversity (Haller et al., 2014; Maccari et al., 2014). In preclinical models, early life stress paradigms have been proposed as a tool to program or bias anxiety traits and, as a consequence, have negative impact in social competence (Tzanoulinou and Sandi, 2017). Indeed, peri-pubertal stress leads to enhanced anxiety (Cordero et al., 2016) and changes in social behavior in adulthood (Haller et al., 2014). Interestingly, play-fighting is a peri-pubertal social behavior which has been linked to aggression in adulthood. Specifically, peri-puberal stress increases play-fighting and increases the chances to display abnormal aggressive behaviors later in adulthood (Papilloud et al., 2018). Importantly, this study also revealed a role of mitochondrial energy balance in regulating stress-induced behaviors, by showing that enhanced play-fighting behaviors following peri-pubertal stress was accompanied by enhanced mitochondrial function in the amygdala.

## 4 Encoding reward-directed behavior under anxiety

In humans, patients suffering from anxiety disorders have impaired decision making and behavioral flexibility (Park and Moghaddam, 2017a). For example, high levels of anxiety are accompanied by difficulty of shifting between strategies in the presence of changes in task demand, and/or are easily distracted by irrelevant stimuli (Eysenck et al., 2007). That inability to change strategies during a task can have detrimental consequences in a person, by affecting personal life and professional performance. The prefrontal cortex (PFC) is a pivotal structure in organizing behavior in a context dependent-manner (Bechara et al., 2000; Miller and Cohen, 2001). Thus, during stress, the PFC controls high order adaptive responses such as choosing optimal behavioral output with an online evaluation of the situation (Moghaddam, 2016).

In rodents, anxiety levels are also related to decreased cognitive flexibility (Park and Moghaddam, 2017a). Recent studies have revealed the functional consequence of anxiety upon PFC activity, by identifying that a negative emotional state elicits sustained reduction of spontaneous firing rate in the dorso-medial PFC (dmPFC) and orbitofrontal cortex (OFC, Park et al., 2016b). This hypofrontality, and specifically the reduced activity in the dmPFC, is linked to decreased behavioral flexibility in the set-shifting task (Park et al., 2016b). In this task the subjects learn an instrumental behavioral response based on two different rules that sit on two different dimensions (for example: shape and color). The ability to switch between rules to maximize the profit (number of rewards) is PFC-dependent. The ventral tegmental area (VTA) is also a critical component of reward-guided behavior, and together with the PFC, has been proposed as a circuit underlying decisions during reward-seeking under punishment. VTA neurons are a key component of the reward circuit (Morales and Margolis, 2017; Wood et al., 2017) and their projections to the mPFC are involved in regulating mood and emotional states (Lammel et al., 2014).

Park and Moghaddam designed and developed a task to study the dynamics of reward-based behavior while risking potential punishment (Park and Moghaddam, 2017b). The task was designed to link instrumental action to reward, but at the same time, the same action will be followed by a punishment with varying probabilities. This task aims to recreate an environment where uncertainty of a negative outcome induces anxiety. The probability of getting punished affects behavioral performance by increasing the variability of the time to react, suggesting a transitory anxiety state caused by the possibility of being punished. Single-unit recordings from both dopaminergic (DA) VTA neurons, and mPFC neurons, during this task revealed that their firing rate around the motor response (time surrounding the action execution) is correlated with the punishment risk, suggesting both neural populations encode the risk probability. Even though both VTA-DA and non-DA neurons, as well as mPFC neurons, showed punishment risk encoding responses, VTA-DA neurons showed a higher temporal resolution around the action time than mPFC neurons, which showed a more diffuse response around the action window. Interestingly, the single-unit recordings did not correlate with the behavioral changes during the task, and a more detailed analysis revealed that the variability in the reaction time was related to a circuit dynamic. The correlation of the firing of VTA and mPFC neurons with the reaction time was evident only in the riskiest part of the session, when the probability to be punished was higher.

At the network level, the synchronicity of the theta oscillations is important to coordinate groups of neurons to complete a behavioral response (Akam and Kullman, 2010; Buschman et al., 2012). Interestingly, during non-punished trials, the oscillations that emerged in the VTA and mPFC are in the theta range (around 8 Hz, Park and Moghaddam, 2017b). Moreover, increased probability of punishment decreases the oscillations in both areas and weakens synchrony within and between both structures.

These studies revealed that synchrony between the VTA and PFC decreases with punishment probability, suggesting that VTA-PFC network encodes punishment risk (Fig. 2C). Under normal conditions, VTA drives oscillation synchronicity between both regions exerting a bottom-up control of the network. When there is risk of punishment, this bottom-up network control is diminished. Transient anxiety may, therefore, affect behavior in a reward-based task by disrupting the VTA-PFC functional circuit.

## 5 Role of hypothalamic corticotropin (PVN-CRH) neurons in stress-related behaviors

Although the relationship between stress and anxiety is bidirectional, the influence of stress as a risk factor for anxiety disorders has been extensively studied (Armario et al., 2008; Tye and Deisseroth, 2012). Mapping of neural circuits involved in stress-induced anxiety have mostly revolved around structures of the amygdala and extended amygdala (Davis et al., 2010; Gross and Canteras, 2012). Surprisingly, relatively less attention has been directed towards the paraventricular nucleus of the hypothalamus (PVN), which is a crucial component of the visceral stress response. The PVN receives and integrates information about the stressor and controls the endocrine, behavioral and autonomic response to stress (Denver, 2009; Herman et al., 2003; Ulrich-Lai and Herman, 2009). Specifically, parvocellular neurosecretory cells release corticotropin-releasing hormone (CRH) into the anterior pituitary that stimulates the synthesis and release in the blood stream of adrenocorticotropic hormone (ACTH). Once ACTH reaches the adrenal glands it stimulates the release of glucocorticoid (Denver, 2009; Herman et al., 2003; Ulrich-Lai and Herman, 2009). In the past five years, the classical view of PVN-CRH neurons has been challenged by demonstrations that these neurons also control multiple and complex behaviors that are linked to stress (De Marco et al., 2016; Füzesi et al., 2016; Sterley et al., 2018; Zhang et al., 2017). These observations open an exciting line of research to investigate how this specific set of neurons can drive stress-induced behavioral alterations.

**Fig. 3:**
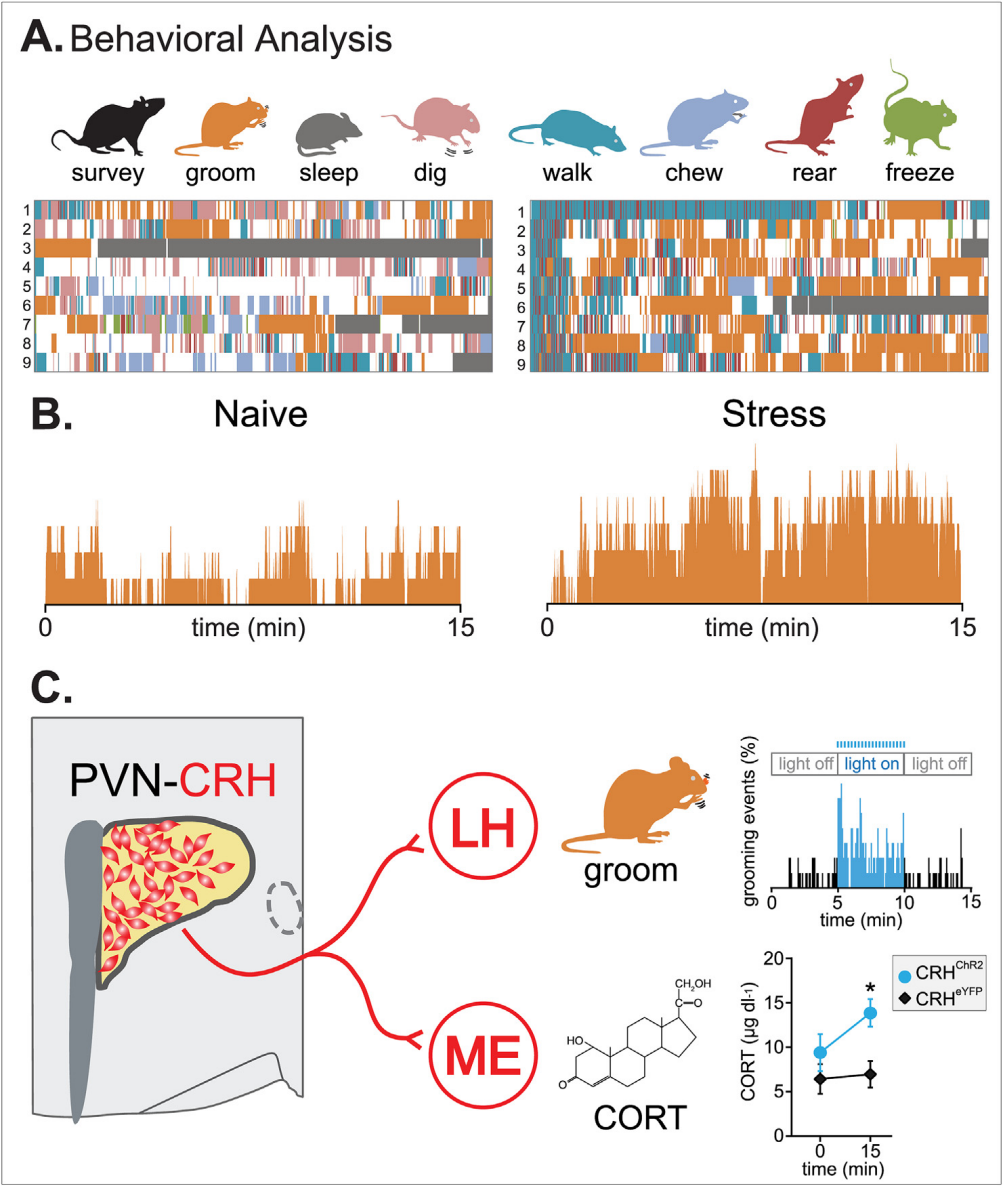
**PVN-CRH neurons coordinate behavioral patterns after stress***(adapted from*Füzesi et al., 2016*)*. **A.** Behavioral quantification of mice in their home-cage, before (naïve) and immediately after a footshock (stress). Eight distinct behaviors are prominent. Each row represents one mouse. **B.** Histogram of grooming behavior at each time-point for naïve and stress mice. **C.** Schematic of *in vivo* photostimulation of PVN-CRH to LH projections (20 Hz, 5 min) increases grooming time and stimulated CORT release. PVN: paraventricular nucleus of the hypothalamus CRH: corticotropin-releasing hormone LH: lateral hypothalamus ME: median eminence CORT: corticosterone.

The standard methods to study anxiety in rodents have relied on established laboratory tests such as the elevated plus maze. However, over the last decade, an increasing number of studies have focused on developing new tools of behavioral analysis that are less invasive, require less subjective interpretations, and harken back to original ethological approaches (Lezak et al., 2017). One of these approaches relies on monitoring freely-behaving mice in their home-cage environment without any intervention (Fig. 3A). Multiple behaviors such as grooming, freezing, rearing or surveying can be detected. Importantly, these behaviors show a specific but flexible temporal distribution. Using this approach, it was shown that a single stressful experience results in the emergence of an organized and structured behavioral pattern where self-directed behaviors such as grooming become predominant, taking over exploratory behaviors such as locomotion or rearing (Fig. 3B, Füzesi et al., 2016). The role of brainstem in generating stress-induced behavioral responses such as freezing (Deng et al., 2016; Silva and McNaughton, 2019), grooming (Kalueff et al., 2016), defensive behaviors or even anxiety-like behavior (McCall et al., 2015), has been extensively studied over the years (Myers et al., 2017). A recent study from Füzesi et al. (2016) proposes the contribution of a different structure, by revealing a new role for PVN-CRH neurons in coordinating the spontaneous behavioral patterns that emerge after acute stress. Specifically, optogenetic silencing of PVN-CRH activity during the post-stress period reduces grooming in a safe environment. On the contrary, activating PVN-CRH neurons modifies the intensity, but not necessarily the temporal sequence of context appropriate behaviors (Fig. 3C). The behavioral profile that arises after an acute stress experience is context dependent and related to PVN-CRH neuron activity.

Further work on PVN-CRH has revealed that these cells are necessary and sufficient for stress-induced social investigation that is required for the transmission of stress from one individual to another (Sterley et al., 2018). This social transmission of stress results in changes in synaptic function and metaplasticity in the recipient mice, which mirror the synaptic changes observed in stressed mice. This study positions PVN-CRH neurons as a critical node for alarm signal processing and offers a potential explanation for how individuals who have not had a first-hand traumatic experience can develop symptomology consistent with post-traumatic stress disorders (Haugen et al., 2012; Sial et al., 2016).

Recent studies have shown that PVN-CRH neurons are able to respond differentially to stimuli with positive or negative valence (Kim et al., 2019; Yuan et al., 2019). Using *in vivo* fiber photometry to monitor population calcium dynamics, Kim et al. (2019) have shown that the activity of PVN-CRH neurons rapidly increases in response to stimuli with negative valence, and decreases when an appetitive stimulus such as food or social interaction is presented. Moreover, a reward presentation during stress can buffer PVN-CRH activation, and, as a consequence, modify stress-related behaviors, such as grooming, correlate with the activity of those neurons (Yuan et al., 2019). The bidirectional response of PVN-CRH neurons based on stimulus valence opens a new perspective to study stress coping and relieve.

Beyond CRH neurons, a distinct neuronal population in the PVN containing CRH1R has been recently described. This population is 90% GABAergic and has direct synaptic contact with PVN-CRH neurons, and CRH, released from PVN-CRH neurons, modulates signaling between both populations (Jiang et al., 2018; Ramot et al., 2017). This intra-PVN network of CRH1R+ and CRF + neurons has been associated with anxiety-like behavior, as knocking out CRH1R from PVN neurons has anxiolytic effects (Ramot et al., 2017; Zhang et al., 2017).

All these new data are contributing to update the role of hypothalamic neural populations in stress-related behaviors. Specifically, growing evidence implicates PVN-CRH neurons in modulating specific behaviors in a stress-related context. From controlling how an individual responds to stress exposure (Füzesi et al., 2016; Kim et al., 2019; Yuan et al., 2019) to regulating anxiety-like behaviors (Ramot et al., 2017; Zhang et al., 2017), or driving social transmission of stress (Sterley et al., 2018), the PVN-CRH neuron population appears to be a central player linking stress and anxiety.

## 6 From research to clinics: new categorization of post-traumatic stress disorder (PTSD)

The idea that stress and anxiety have segregated neurobiological substrates despite their reciprocal influence has now moved beyond the research field to reach the clinical practice, resulting in changes in diagnostic tools. Specifically, according to the DSM-5 (American Psychiatric Association, 2013) PTSD is not classified as an anxiety disorder anymore, and the new manual categorizes it among the *trauma or stressor-related disorders*. This new category requires explicitly an exposure to a traumatic or stressful event as a diagnostic criterion, and in particular, the PTSD requires a life threatening experience. Reclassifying PTSD from anxiety disorders to this newly created *trauma or stressor-related disorders* category has helped to move the focus away from anxiety, which is now rather considered as a comorbid pathology. The new classification in DSM-5 also emphasizes the abnormal reactivity to a stimulus. Interestingly, an altered “flight” response has been observed in patients with PTSD (Park et al., 2017) and, in the last five years, the interest in how microcircuits controlling threat processing are altered in PTSD has grown considerably. This emergent literature supports the idea that aberrant subconscious threat-related processes are underlying part of the PTSD symptomatology (Lanius et al., 2017). Current data indicates an increased connectivity between areas involved in the innate alarm system, such as the LC, amygdala, hypothalamus, and PFC in PTSD patients (Rabellino et al., 2016). In humans, when a challenge is not life-threating, the subjects choose the most suitable cost-effective strategy to overcome that situation by engaging the vmPFC, medial orbitofrontal cortex (mOFC) and other non-cortical structures including the BLA which is causally involved in valence processing. However, when the level of danger is life threatening and the organism must prepare for a defensive response, subcortical structures, such as the periaqueductal gray area (PAG) and CeA overrule the cognitive control, and guide the behavioral response (Mobbs et al., 2007). The balance between cortical and subcortical systems could also cast out key points to understand some anxiety disorders. Negative emotional states such as anxiety may lead a person to overestimate the possibility of danger, leading to a shift in the balance of these two systems. The dynamics between the cognitive and innate circuitry in response to a challenge may reveal a path to a better comprehension of how stressful experiences influence our emotional state, and in turn, shape our future behaviors.

## 7 Perspective

Although the dissection of multiple brain circuits controlling anxiety- and stress-related behaviors has made substantial advances, our understanding of the neural substrates underlying the interplay between these two psychophysiological responses remains fragmented. Here, we described specific roles of neural populations in the amygdala, nucleus accumbens, prefrontal cortex, hypothalamus and locus coeruleus in emotional valence, stress and anxiety. The work described here is providing foundational knowledge, however future investigation is necessary to unravel the contribution of specific circuits to stress and/or anxiety. Specifically, future work should use activity dependent mapping and recordings of genetically or anatomically defined neural populations during stress exposure and at different levels of anxiety within the same animal. Beyond the technical advances catalyzing our understanding of the neural circuits underpinning stress and anxiety, disentangling them will require the development of new behavioral paradigms in pre-clinical models in order to finely capture the changes of neural coding in these two conditions.

## Conflicts of interest

The authors have no conflict of interest to declare.
